# Variability of large timescale functional networks in patients with disorders of consciousness

**DOI:** 10.3389/fneur.2024.1283140

**Published:** 2024-02-15

**Authors:** Anjuan Gong, Qijun Wang, Qian Guo, Ying Yang, Xuewei Chen, Xiaohua Hu, Ying Zhang

**Affiliations:** ^1^Center for Cognition and Brain Disorders, The Affiliated Hospital of Hangzhou Normal University, Hangzhou, Zhejiang, China; ^2^Hangzhou Normal University School of Nursing, Hangzhou, Zhejiang, China; ^3^Department of Rehabilitation Medicine, Armed Police Corps Hospital of Zhejiang Province, Hangzhou, Zhejiang, China

**Keywords:** disorders of consciousness, EEG, functional connectivity, complex network, assessment of brain function

## Abstract

**Objective:**

Most brain function assessments for disorders of consciousness (DOC) utilized quantified characteristics, measured only once, ignoring the variation of patients' brain states. The study aims to investigate the brain activities of patients with DOC from a new perspective: variability of a large timescale functional network.

**Methods:**

Forty-nine patients were enrolled in this study and performed a 1-week behavioral assessment. Subsequently, each patient received electroencephalography (EEG) recordings five times daily at 2-h intervals. Functional connectivity and networks were measured by weighted phase lag index and complex network parameters (characteristic path length, cluster coefficient, and betweenness centrality). The relative coefficient of variation (CV) of network parameters was measured to evaluate functional network variability.

**Results:**

Functional networks of patients with vegetative state/unresponsive wakefulness syndrome (VS/UWS) showed significantly higher segregation (characteristic path length) and lower centrality (betweenness centrality) than emerging from the minimal conscious state (EMCS) and minimal conscious state (MCS), as well as lower integration (cluster coefficient) than MCS. The functional networks of VS/UWS patients consistently presented the highest variability in segregation and integration (i.e., highest CV values of characteristic path length and cluster coefficient) on a larger time scale than MCS and EMCS. Moreover, the CV values of characteristic path length and cluster coefficient showed a significant inverse correlation with the Coma Recovery Scale-Revised scores (CRS-R). The CV values of network betweenness centrality, particularly of the cento-parietal region, showed a positive correlation with the CRS-R.

**Conclusion:**

The functional networks of VS/UWS patients present the most invariant segregation and integration but divergent centrality on the large time scale networks than MCS and EMCS.

**Significance:**

The variations observed within large timescale functional networks significantly correlate with the degree of consciousness impairment. This finding augments our understanding of the neurophysiological mechanisms underpinning disorders of consciousness.

## Introduction

The human brain consists of approximately one hundred billion neurons interconnected by thousands of trillions of synapses. Its intricacy extends beyond mere numerical metrics, encompassing the hierarchical arrangement of connections across various scales. The configurations of these connections give rise to functions, including consciousness (1, 2). Disorders of consciousness (DOC) include complicated clinical symptoms (3). Patients can be categorized into a vegetative state/unresponsive wakefulness syndrome (VS/UWS) or a minimally conscious state (MCS) based on their responsiveness to external stimuli (4). UWS patients maintain behavioral arousal but lack awareness (5, 6). MCS patients show signs of varying, yet reproducible, remnants of non-reflex behaviors (7). Patients who have regained accurate communication and/or functional use of objects are defined as emergence from MCS (EMCS) patients. Recently, with the rapid development of techniques, studies have attempted to uncover the mystery of consciousness injury with different perspectives, such as neuro-image (8, 9), neuro-spectroscopy (10, 11), and electro-neurophysiology (12, 13). However, patients with DOC underwent different types of brain injury and generated significant individual differences, such as lesion size and location. These etiological variations pose challenges to group studies on DOC patients. Thus, far, it is still insufficient to recognize the brain states of patients with DOC.

Electroencephalography (EEG) is commonly used in assessing brain activities in patients with brain diseases. Existing research has leveraged EEG to evaluate various aspects in patients with DOC, including the resting-state brain condition (13), event-related brain activities (14), sleeping (15), and neural responsiveness (16). Of these, resting-state EEG is particularly informative in monitoring and capturing the characteristics of patients' foundational neural activities (17). It provides several dimensionalities to describe the brain conditions of patients with DOC, with the functional network emerging as a key dimension in resting-state EEG analysis. It quantifies the capacity of information interaction within different neural units of the brain. Studies have revealed that information interaction and integration are important characteristics of human consciousness (18). Therefore, the functional network is intimately linked to consciousness injury and recovery of patients with DOC (19, 20). Studies have revealed that patients with preserved functional networks are more likely to exhibit consciousness signs (21, 22). Compared with MCS patients, VS/UWS patients have always shown significantly worse functional networks (e.g., decreased network interaction) (23). Moreover, the functional network (alpha participation coefficients) has shown a close association with patients' brain metabolism (24) and sensitivity to brain intervention (25). Studies show that a functional network measurement is a critical approach for assessing the brain states of patients with DOC and exploring the underlying mechanisms of conscious injury and rehabilitation.

Studies have shown that the brain state of patients with DOC tends to vary (26–28). Such a conscious variation is one of the critical impacts of misdiagnosis using behavioral scales (27). Notably, the Coma Recovery Scale-Revised (CRS-R) assessment yields inconsistent results, with patients performing better in the morning than in the afternoon, even on the same day (29). This variation has also been evidenced in EEG studies leading to inconsistent diagnoses when comparing results from the CRS-R and a combination of transcranial magnetic stimulation and EEG (30). Moreover, the same patients also showed various perturbational complexity indexes at different time points (31). Periodic variations (roughly every 70 min) have been reported in MCS patients using resting-state EEG, with no distinct periodicity in UWS patients (32). Cai et al. (33) found a flexible network (transition of community assignment) of DOC patients on the second scale, suggesting that the presence of various brain conditions is a foundational property in DOC patients. However, despite these findings, brain network studies typically rely on single-time measurements of quantified characteristics, overlooking potential network variations in DOC patients, especially on a larger time scale. This study hypothesized that the large timescale variation of brain networks might be correlated with the extent of consciousness injury.

Based on the above considerations, this study measured the functional connectivity and networks of patients with DOC on a large time scale and explored the variability of these characteristics.

## Methods and materials

### Patients

Forty-nine patients (age: 54 ± 12 years; men: *n* = 29 and women: *n* = 20) diagnosed with DOC (EMCS, *n* = 10; MCS, *n* = 16; VS/UWS, *n* = 23) were enrolled in this study. The statistical characteristics of the patients are listed in Table 1. The details of the patients are given in Supplementary Table 1. All the patients had no history of epilepsy, pacemakers, aneurysm clips, neurostimulators, or brain/subdural electrodes. Patients in the study had not received treatments with zolpidem, modafinil, amantadine, midazolam, or baclofen. All the patients were free from acute medical complications (e.g., acute pneumonia) for at least 2 weeks prior to the commencement of the study. The patients had a clear, long-lasting wakeful state time in the day, as reported daily by their nurses. During the experiment, all the patients received consistent routine medications (physical treatment) and nursing (including nutrition and massage) within a controlled environment of room temperature and light. Caregivers provided written informed consent for participation. According to the Declaration of Helsinki, this study was approved by the ethics committee of the Affiliated Hospital of Hangzhou Normal University.

**Table 1:** Characteristics of the patients.

**Characteristic**	**Value**
Age	54 ± 12 years
Gender	Male = 29; Female = 20
Time from onset of acute brain injury	3.3 ± 2 months, range: 2–12
**Cause of acute brain injury: (number of patients)**	
Cardiac arrest	6
Intracerebral hemorrhage	13
Traumatic brain injury or subdural hematoma	18
Subarachnoid hemorrhage	12
**Coma recovery scale-revised score**	
Vegetative state	*n* = 23, score: 5.0 ± 1.2
Minimally conscious state	*n* = 16, score: 9.2 ± 2.8
Emerge minimally conscious state	*n* = 10, score: 20.6 ± 2.6

### Behavioral evaluation

A trained neurologist conducted a CRS-R (34) evaluation for 1 week, comprising at least five assessments during the afternoon sessions on separate days. The CRS-R scale includes six items that test auditory, visual, motor, oromotor, communication, and arousal functions. CRS-R is both quantitative (scores range from 0 to 23) and qualitative, with some key behaviors defining different states of consciousness (coma, VS/UWS, MCS, or EMCS). After repeated CRS-R tests, the neurologist used the highest scores to diagnose the patients' states: VS/UWS, MCS, and EMCS.

### EEG recording and pre-processing

This study conducted EEG recordings 1 to 2 days after the diagnosis assessment. Each patient received EEG recordings five times at different time points in the day (T1, 9:00; T2, 11:00; T3, 13:00; T4, 15:00; T5, 17:00), each lasting for 15 min. Before each recording, a CRS-R arousal facilitation protocol was performed on the patients to maintain their arousal. No nutrition was given at least half an hour before and during the EEG recordings. All the patients lay on the bed and remained in an arousal state (open eyes) during the EEG recordings. The patients were monitored for possible EEG signs of drowsiness and sleep onset (i.e., an increase in tonic theta rhythms and sleep spindles). The recording would be momentarily interrupted to apply the CRS-R arousal facilitation protocols in case of drowsiness or sleep.

Each EEG recording was performed using a 32-channel EEG recorder (BrainAmp 32 MRplus, BrainProducts) under consistent conditions. The EEG cap includes 30 data recording channels (Fp1, Fp2, F7, F3, Fz, F4, F8, FC5, FC1, FC2, FC6, T7, C3, Cz, C4, T8, TP9, CP5, CP1, CP2, CP6, TP10, P7, P3, Pz, P4, P8, O1, Oz, and O2), a reference electrode (location at FCz), and a ground (location at AFz) electrode according to the 10/10 international system. The equipment used sintered Ag/AgCl pin electrodes. We set a bandpass filtered at DC to 1,000 Hz in the recorder, with the EEG signals digitized at a sampling rate of 2.5 kHz. The skin/electrode impedance was maintained below 5 kΩ.

An offline pre-processing was performed using EEGLAB 12.0.2.5b, running in a MATLAB environment (version 2013b, MathWorks Inc., Natick, USA). The EEG data were downsampled to 500 Hz and bandpass filtered (1–45 Hz). Bad channels were automatically detected and rejected using statistical methods based on the spectrum and kurtosis distribution (35). The missing channels were interpolated by superfast spherical interpolation. The EEG signals were divided into non-overlapping epochs (10 s each) for pre-processing. Bad trials were further identified and rejected by combining the automatic probability distribution measurement and visual inspection. The data would be rejected if the number of bad epochs was over 30%. Moreover, a fast, independent component analysis (using the Tanh contrast function) was conducted. Each component's topography and spectrum were visually inspected to identify and remove artifact components, including eye movement, heartbeat, and muscle activities (Supplementary Figure 1). Finally, after aligning to a common average reference, we obtained clean epochs from each patient, ready for network analysis.

### Brain network analysis

The weighted phase lag index (wPLI) is a conservative measure of phase synchronization between electrodes, allowing investigation of phase synchronization characteristics while negating the adverse impact of volume conduction (36). The wPLI was computed for each EEG channel across the other channels based on the following equation (36):


$$\begin{array}{l} wPLI_{i,j}=\frac{\left|E\left\{ℑ\left\{X_{i}X_{j}^{*}\right\}\right\}\right|}{E\left\{\left|ℑ\left\{X_{i}X_{j}^{*}\right\}\right|\right\}}=\frac{\left|E\left\{|ℑ\left\{X_{i}X_{j}^{*}\right\}|sgn\left[ℑ\left\{X_{i}X_{j}^{*}\right\}\right]\right\}\right|}{E\left\{|ℑ\left\{X_{i}X_{j}^{*}\right\}|\;\right\}}, \end{array}$$

where i and j are the channel indices, *X*_*i*_ is the time-frequency spectrum of channel i, $X_{j}^{*}$ is the complex conjugate of *X*_*j*_, $ℑ\left\{X_{i}X_{j}^{*}\right\}$ is an imaginary section of the cross-frequency spectra $X_{i}X_{j}^{*}$, *E*{.} is the expected value operator, and *sgn*{.} is the sign function operator. In practice, we used the Hilbert transform to obtain the time-frequency spectrum of signals with frequency band at 1–45 Hz, the other parameters included window length of 2s and overlapping of 1s. The functional connectivity patterns were obtained after the wPLI measurement, each node's degree representing the mean intensity of its connectivity with other nodes. Each connectivity pattern (T1, T2, T3, T4, and T5) from the epochs of each EEG recording was consolidated. To quantify the network variability, we measured the similarity of the connectivity patterns using normalized mutual information (NMI) (37–39) for each pairwise neighboring epoch. NMI is a metric commonly used in information theory and data clustering to quantify the degree of similarity between two datasets based on the following equation. It measures the mutual dependence between the datasets while accounting for differences in their sizes. The normalization aspect ensures that the value falls within a standardized range, facilitating comparisons across different datasets or clustering algorithms.


$$\begin{array}{l} NMI\left(A,B\right)=\frac{-2\sum_{i=1}^{C_{A}}\sum_{j=1}^{C_{B}}N_{ij}\log\left(\frac{N_{ij}N}{N_{i}N_{j}}\right)}{\sum_{i=1}^{C_{A}}N_{i}\log\left(\frac{N_{i}}{N}\right)+\sum_{j=1}^{C_{B}}N_{j}\log\left(\frac{N_{j}}{N}\right)}\; \end{array}$$

where A and B are different patterns, *C*_*A*_ is the number of modules in pattern A, and *C*_*B*_ is the number of modules in pattern B. N shows the number of nodes, which is the same in both patterns, and *N*_*ij*_ is the overlap between A's module i and B's module j, i.e., the number of nodes that the modules have in common; *N*_*i*_ is the total number of nodes in A's module i; *N*_*j*_ is the total number of nodes in B's module j. The NMI ranges from 0 to 1, where 0 signifies that the patterns are totally independent and 1 signifies that they are identical (40).

Each pairwise neighboring pattern generated an NMI value (a larger value indicates more similarity). The global explained variance (GEV) (41) was used to measure the total variability explained by an average pattern among the connectivity patterns, with a higher GEV implying less variability. In this way, the GEV values and the average of the NMI values could index the variability of the functional connectivity patterns for the patients.

Brain networks are invariably complex, share a number of common features with networks from other biological and physical systems, and may hence be characterized using complex network methods. Complex network analysis describes important properties of complex systems by quantifying topologies of their respective network representations (1). The average shortest path length between all pairs of nodes in the network is known as the characteristic path length of the network (42) and is the most commonly used measure of functional integration.


$$\begin{array}{l} L=\frac{1}{n}\underset{i\in N}{\sum }L_{i}=\frac{1}{n}\underset{i\in N}{\sum }\frac{\sum_{j\in N,j\neq i}d_{ij}}{n-1}\; \end{array}$$

where L_i_ is the average distance between node *i* and all other nodes, and *d*_*ij*_ is the shortest path length (distance) between node *i* and *j*. Characteristic path length reflects the overall efficiency of information integration between different brain regions. Locally, the fraction of triangles around an individual node is known as the cluster coefficient and is equivalent to the fraction of the node's neighbors that are also neighbors of each other (42).


$$\begin{array}{l} C=\frac{1}{n}\underset{i\in N}{\sum }C_{i}=\frac{1}{n}\underset{i\in N}{\sum }\frac{2t_{i}}{k_{i}\left(k_{i}-1\right)}\; \end{array}$$

where *C*_*i*_ is the clustering coefficient of node *i* (*C*_*i*_ = 0 for *k*_*i*_ < 2), and *t*_*i*_ is the number of triangles around node *i*. It reflects the information processing efficiency of the local brain area of the brain network. Betweenness centrality is defined as the fraction of all shortest paths in the network that pass through a given node (43).


$$\begin{array}{l} b_{i}=\frac{1}{\left(n-1\right)\left(n-2\right)}\underset{\begin{array}{c} h,j\in N,\\ h\neq j,h\neq i,j\neq i, \end{array}}{\sum }\frac{\rho_{hj}\left(i\right)}{\rho_{hj}}\; \end{array}$$

where ρ_hj_ is the number of shortest paths between h and j, and ρ_hj_ (i) is the number of shortest paths between h and j that pass through i. Betweenness centrality can find the vertices that have a great influence on the information flow of the graph, and the larger the value, the greater the influence of the vertices on the information flow.

The connectivity patterns can be modeled as networks, with the electrodes as nodes, and the *wPLI*_*i,j*_ values denote the connection strength. The connectivity patterns were submitted to the graph theory algorithms implemented in the Brain Connectivity Toolbox (44) to calculate the metrics that captured the key topological characteristics of the graphs, such as the micro-scale cluster coefficient (segregation), the macro-scale characteristic path length (integration), and betweenness centrality (centrality). There is no thresholding involved in the computation of the key topological characteristics of the graphs. It is worth noting that for each EEG recording (T1-T5), we first calculated the functional connectivity and corresponding network characteristics of each epoch (10s) and then took the average across all epochs. Furthermore, for each patient, we measured the relative coefficient of variation (CV) (ratio of the standard deviation to the mean) among the network parameters obtained from the five recordings. The CV values of the network parameters were used to quantify the variability of the functional networks.

### Statistical analyses

Statistical analyses were performed using SPSS version 20.0 (SPSS Inc., Chicago, IL, USA). The significance of the NMI and GEV values between the pairwise diagnosis groups was tested by the Mann-Whitney U test. Bonferroni corrections were conducted after multi-comparison (three comparisons VS/UWS vs. MCS, VS/UWS vs. EMCS, and MCS vs. EMCS). The Mann-Whitney U test combined with the Bonferroni correction (three comparisons) was used to test the significance of average degree values, characteristic path length, cluster coefficient, and betweenness centrality in each pairwise diagnosis group. The significance of the CV values of degree value or betweenness in diagnosis group contrast was tested by the Friedman test in each electrode. The false discovery rate correction (Q = 0.05) was used to correct the *p*-values after multi-comparisons (30 comparisons for each diagnosis group). The CV values of functional networks (characteristic path length, cluster coefficient, and betweenness centrality) were compared between the pairwise diagnosis groups using the Mann-Whitney U test with Bonferroni corrections. The Kendall correlation measured correlational analyses of the CV values with the patients' CRS-R. All the presented *p*-values were obtained after the multi-comparison correction.

## Results

**Figure 1:**
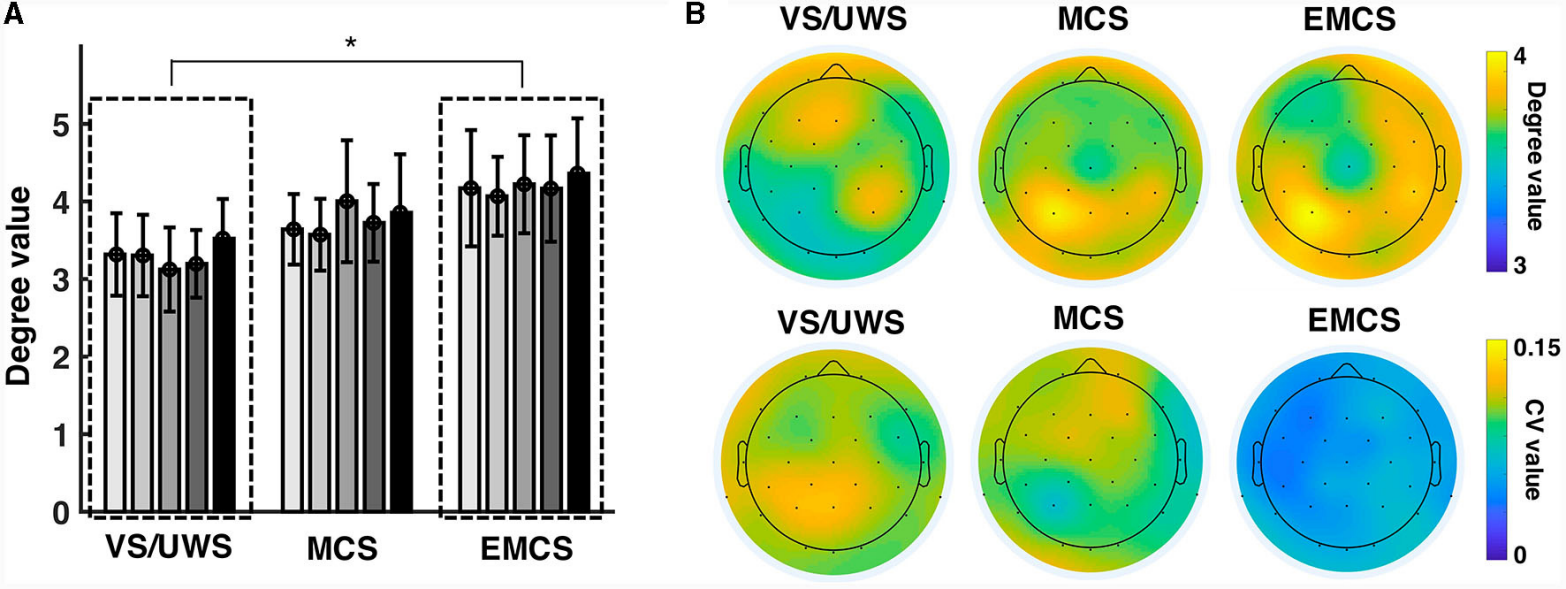
Similarity of the connectivity patterns in vegetative state/unresponsive wakefulness syndrome (VS/UWS), minimal conscious state (MCS), and emergence from MCS (EMCS). **(A)** The bars show the average degree values of the patients at five-time recordings (T1, T2, T3, T4, and T5). The Kruskal-Wallis test was used to test the significance of the average degree (among all five recordings) across the diagnosis groups (VS/UWS, MCS, and EMCS). The pairwise test used the Mann-Whitney U test with Bonferroni correction. *Significant difference, *p* < 0.05. **(B)** The topographies show the degree values averaged among the five recordings of the electrodes (upper) as well as their CV values (lower).

The average degree (among all five recordings) showed significance (*p* = 0.020, Kruskal-Wallis test) between the diagnosis groups (three levels: VS/UWS, MCS, and EMCS) (Figure 1A). The pairwise tests showed a significantly lower (*p* = 0.016, Mann-Whitney U test) average degree value of VS/UWS (mean ± 1SD, 3.291 ± 0.512) than EMCS (mean ± 1SD, 4.202 ± 0.659) patients. There was no significant difference between the MCS and VS/UWS. On the electrode level, the parietal region of MCS and EMCS patients demonstrated higher degree values but lower coefficient of variation (CV) values than those of VS/UWS (Figure 1B).

**Figure 2:**
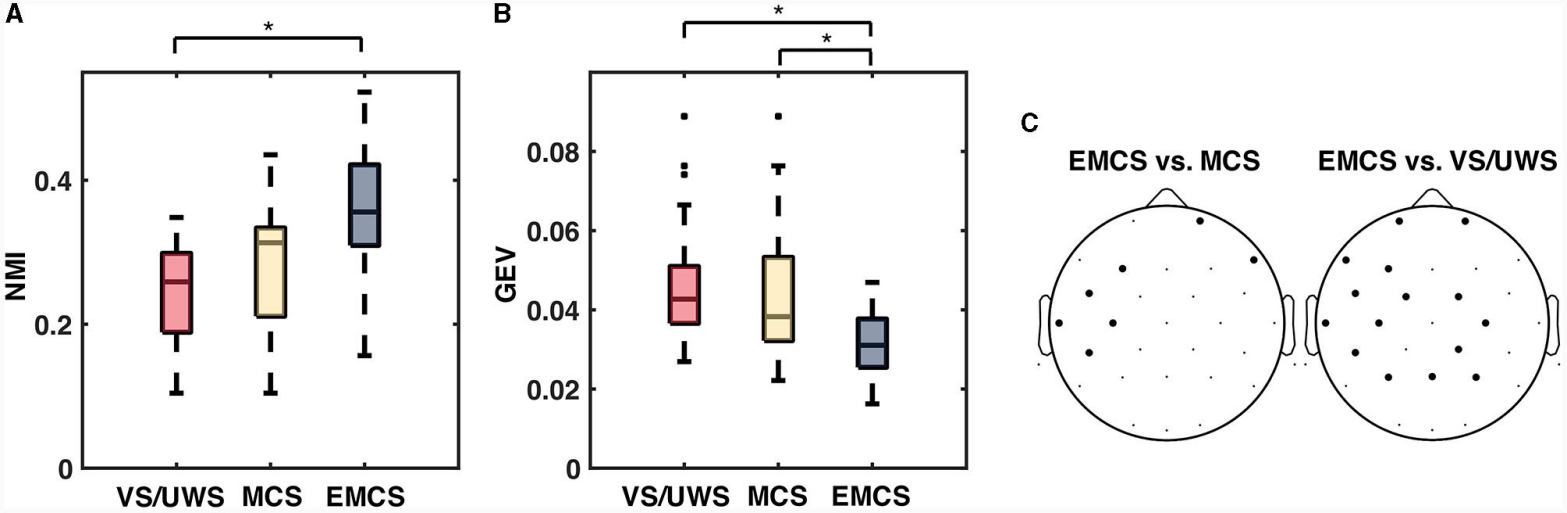
The significant difference of normalized mutual information (NMI) and global explained variance (GEV) between vegetative state/unresponsive wakefulness syndrome (VS/UWS), minimal conscious state (MCS), and emergence from MCS (EMCS). **(A)** Box plots of normalized mutual information (NMI), which measured the similarity of each pairwise neighboring recording. **(B)** Box plots of global explained variance (GEV), which measured how much the average connectivity pattern explained the variance among the patterns from all epochs. The Kruskal-Wallis test was used to test the significance among the diagnosis groups on each time scale. The pairwise test used the Mann-Whitney U test with Bonferroni correction. **(C)** Electrodes (bold dots) with significantly different (Friedman test with false discovery rate correction) CV values of degree between EMCS vs. MCS and EMCS vs. VS/UWS. *Significant difference after correction, *p* < 0.05.

The NMI indexed the connectivity similarity of the pairwise neighboring recording. The NMI values of the EMCS patients (median ± IQR, 0.356 ± 0.112) were significantly higher (*p* = 0.010, Mann-Whitney U test) than those of the VS/UWS (median ± IQR, 0.259 ± 0.110) patients (Figure 2A). There was no significant difference between MCS and the other two groups (VS/UWS, EMCS patients). In the variability measurement (Figure 2B), the average connectivity patterns explained more variability with significantly lower GEV values in EMCS patients (median ± IQR, 0.031 ± 0.012) than in MCS (median ± IQR, 0.038 ± 0.021) (*p* = 0.020, Mann-Whitney U test) and UWS patients (median ± IQR, 0.043 ± 0.014) (*p* = 0.020, Mann-Whitney U test). On an electrode level, EMCS showed significantly lower (*p* < 0.05, Friedman test with FDR correction) degree CV values than MCS in 7/30 electrodes than VS/UWS in 15/30 electrodes (Figure 2C).

**Figure 3:**
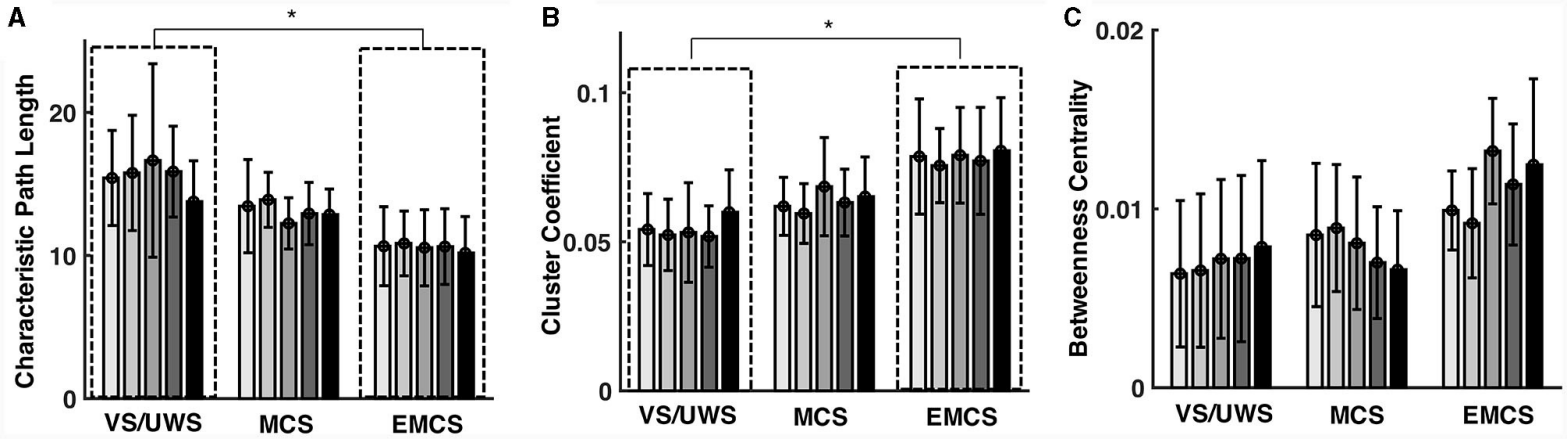
Characteristic path length (CPL) cluster coefficient (CC) and betweenness centrality (BC) of the functional network in vegetative state/unresponsive wakefulness syndrome (VS/UWS), minimal conscious state (MCS), and emergence from MCS (EMCS). The bars show the patients' path length values **(A)**, cluster coefficient values **(B)**, and betweenness centrality values **(C)** in each EEG recording. Significance tests were conducted using the Mann-Whitney U tests with Bonferroni correction. *Means *p* < 0.05.

Figures 3A–C show the path length, cluster coefficient values, and betweenness centrality of all the patients in each EEG recording. VS/UWS patients showed significantly higher (*p* = 0.010, Mann-Whitney U test) (Figure 3A) characteristic length path (mean ± 1SD, 15.493 ± 4.031 vs. 10.620 ± 2.545) and lower (*p* = 0.015, Mann-Whitney U test) cluster coefficient (mean ± 1SD, 0.054 ± 0.013 vs. 0.077 ± 0.017) (Figure 3B) than EMCS patients. However, betweenness centrality did not show significant differences in VS/UWS, MCS, and EMCS.

**Figure 4:**
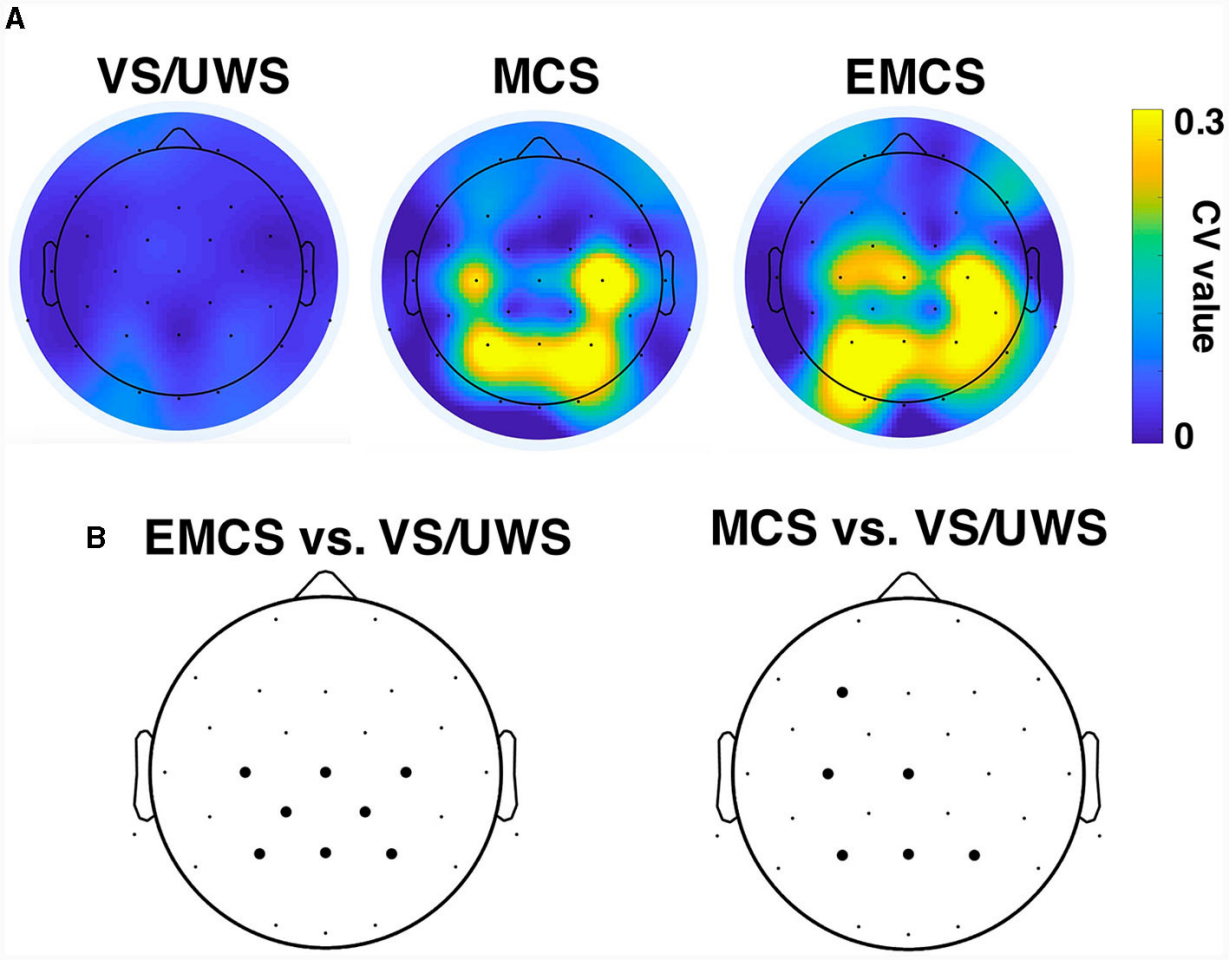
Betweenness centrality of the functional network in vegetative state/unresponsive wakefulness syndrome (VS/UWS), minimal conscious state (MCS), and emergence from MCS (EMCS). **(A)** CV values of betweenness centrality of each electrode in VS/UWS, MCS, and EMCS patients. **(B)** Electrodes (bold dots) with significantly different (Friedman test with false discovery rate correction) CV values of betweenness centrality compared to EMCS vs. VS/UWS and MCS vs. VS/UWS.

VS/UWS patients showed significantly lower average betweenness than MCS (mean ± 1SD, 0.045 ± 0.025 vs. 0.098 ± 0.042, *p* < 0.001, Mann-Whitney U test) and EMCS patients (mean ± 1SD, 0.045 ± 0.025 vs. 0.096 ± 0.049, *p* < 0.001, Mann-Whitney U test). The betweenness showed higher CV values in centro-parietal electrodes in MCS and EMCS patients than in VS/UWS patients (Figure 4A). Notably, the VS/UWS patients had significantly lower (*p* < 0.05, Friedman test with FDR correction) CV values of betweenness than EMCS in Cz, C3, C4, CP1, CP2, Pz, P3, and P4 electrodes and significantly lower CV values than MCS in F3, Cz, C3, Pz, P3, and P4 (Figure 4B).

**Figure 5:**
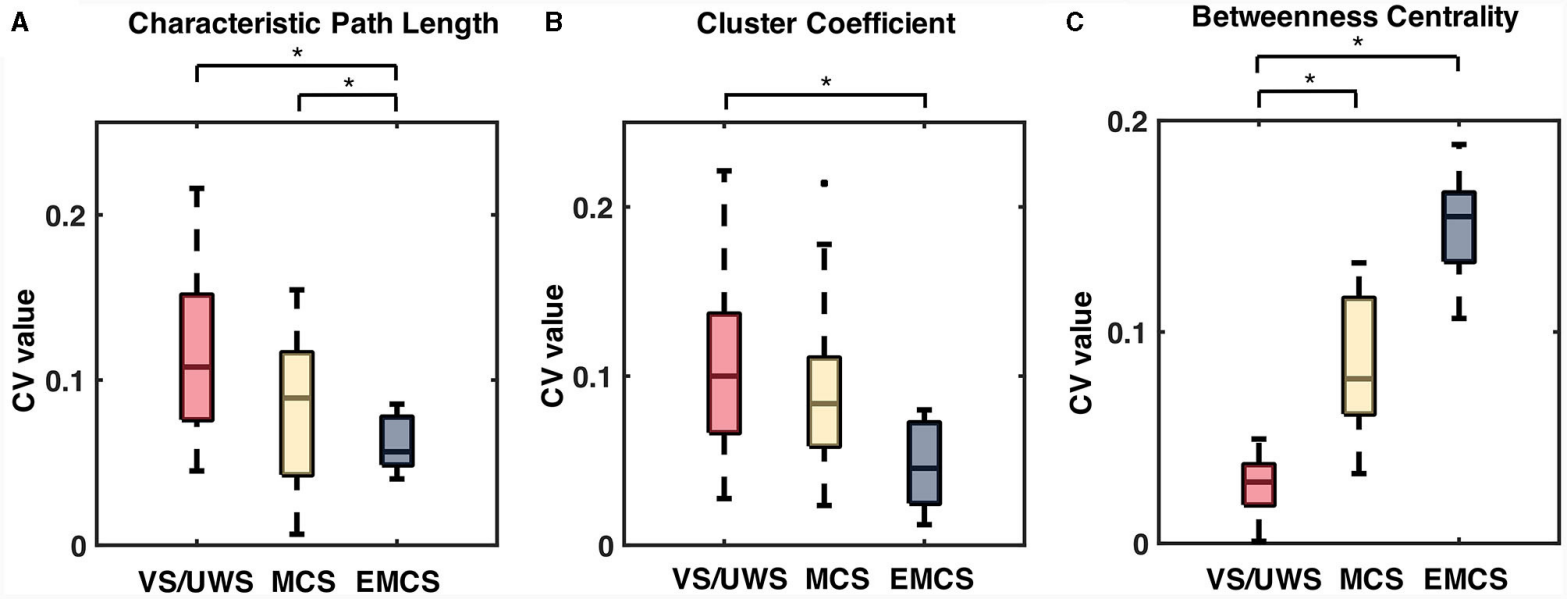
The significant difference in CV values of network characteristics, including characteristic path length (CPL), cluster coefficient (CC), and betweenness centrality (BC) of the patients. The box plots show the CV values of the characteristic path length **(A)**, cluster coefficient **(B)**, and betweenness centrality values **(C)** in patient groups. Significance tests were conducted using the Mann-Whitney U tests with Bonferroni correction. *Means *p* < 0.05.

**Figure 6:**
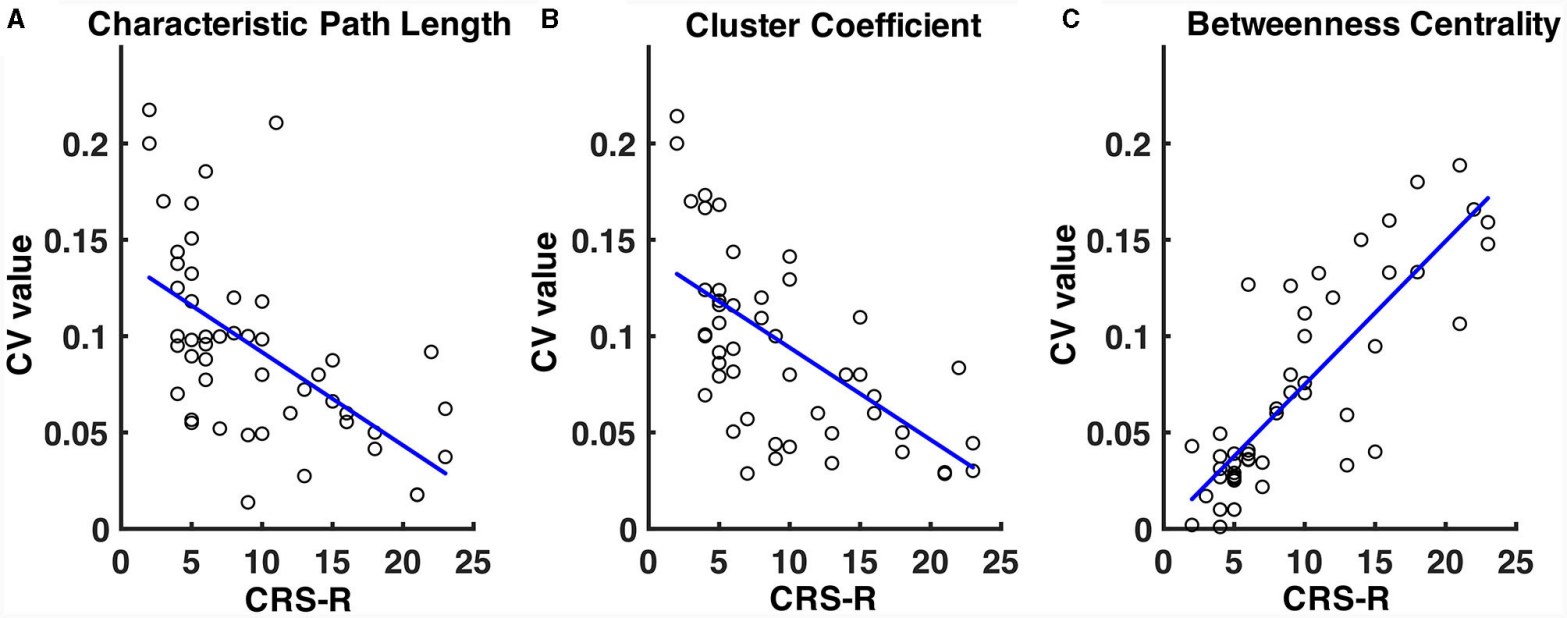
Correlation of CV values of path length **(A)**, cluster coefficient values **(B)**, and betweenness centrality **(C)** with the patients' CRS-R scores. The CRS-R scores were the highest of the patients in a 1-week diagnosis. The blue lines show the linear fitting of the CV values and the CRS-R scores.

The CV values of the characteristic path length of the EMCS patients were significantly lower than those of the VS/UWS (median ± IQR, 0.054 ± 0.026 vs. 0.101 ± 0.054, *p* = 0.009, Mann-Whitney U test) and MCS (median ± IQR, 0.054 ± 0.026 vs. 0.080 ± 0.067, *p* = 0.040, Mann-Whitney U test) groups (Figure 5A). The patients' CRS-R scores showed a significantly negative correlation (*r* = −0.46, *p* < 0.001) (Figure 6A). The CV values of cluster coefficient from EMCS patients were significantly lower than those of the VS/UWS (median ± IQR, 0.055 ± 0.036 vs. 0.115 ± 0.0734, *p* = 0.009, Mann-Whitney U test), but not than those of MCS patients (median ± IQR, 0.055 ± 0.036 vs. 0.091 ± 0.0788, *p* > 0.05, Mann-Whitney U test) (Figure 5B). The patients' CRS-R scores showed a negative correlation (*r* = −0.47, *p* < 0.001) (Figure 6B). Notably, the VS/UWS patients had significantly lower (*p* < 0.05, Friedman test with FDR correction) CV values of betweenness centrality than the MCS and EMCS groups (Figure 5C). Additionally, a strong positive correlation (*r* = 0.605, *p* < 0.001) was observed between the CRS-R scores of the patients and the betweenness CV values (Figure 6C).

## Discussion

This study recorded EEG repeatedly (five times at 2-h intervals in a day) from patients with DOC and investigated the variability of functional connectivity patterns and networks on large time scales. The DOC patients showed different network variabilities between VS/UWS and MCS. Specifically, the VS/UWS patients demonstrated greater network variability than the MCS and EMCS patients. This variability was attributed to similarity, GEV measurement of connectivity patterns, and network integration and segregation of CV values.

Naro et al. (45) addressed functional connectivity dynamics in patients with DOC. Although no quantified analysis was conducted, we observed significant functional connectivity dynamics in the MCS patients, a phenomenon absent in the VS/UWS group. It revealed such a phenomenon in the instantaneous time scale (millisecond). However, our findings showed converse results that VS/UWS had more “dynamic” networks than MCS on a large time scale, reflecting the macroscopic transitions of brain states. The network dynamics summarily presented contradicting properties of DOC patients between the short (millisecond and second) and large time scales (hour). The VS/UWS patients showed a relatively more monotonic functional network of instantaneous neural activities than the MCS patients. However, their functional network was variable over extended periods. Piarulli et al. (32) continuously monitored the neural activities of patients with DOC for a prolonged duration (4 h) and found that the MCS patients had a relatively stable regularity of brain activities that could not be found in the VS/UWS patients. However, Piarulli et al. did not measure the network features but measured the signal complexity at the electrode level, which reflected the information capacity of the underlying local brain region. In methodology, the regularity of local regions makes it easier to form stable coupling patterns in networks. By contrast, the randomness of EEG in local regions corresponds to the inordinate global coupling patterns measured by functional connectivity. The observed variability in functional connectivity in VS/UWS patients aligns with their irregular EEG patterns at the electrode level. This confirms that VS/UWS patients exhibit greater network variability over longer time scales, corroborating our study findings.

The findings indicated that the patients with worse behavioral scores exhibited more variable functional networks on a large time scale. The variability of large-scale networks in DOC patients has not been previously explored. It is important to clarify that heightened network variability in VS/UWS patients does not directly equate to increased brain activity. Although the VS/UWS patients showed a more variable network on the large time scale, the average degree value of the network was still significantly lower than that of the MCS and EMCS patients. Therefore, network variability could reflect the widely suppressed cortical activities of VS/UWS patients. The large-scale impairment of neural circuits blocked the information interaction among local neuro groups, which caused more neuro islands and decreased information integration in the brain (46). The independent cortical “islands” generated divergent and disorganized brain networks in VS/UWS. By contrast, although variable, MCS patients maintained a degree of network organization, as indicated by high cluster coefficients and betweenness centrality comparable to those in EMCS. From the view of an integral brain state, the excessively separated neural activities could not generate coordinating and stable coupling patterns for a long time, which is represented by variable functional networks on a larger time scale. VS/UWS brains had less organized brain activities with brain regions, which rendered the functional networks dispersed. Higher characteristic path length, lower cluster coefficient, and lower betweenness centrality values in VS/UWS could be revealed more than in MCS or EMCS. Thus, the large time networks, which express the basic brain conditions, were variant because of the dependent activities of a large number of islands in VS/UWS brains. This hypothesis, however, necessitates substantiation through additional research.

Important brain regions (hubs) often interact with many other regions, facilitate functional integration, and play a key role in network resilience to insult. Measures of node centrality (i.e., betweenness centrality) variously assess the importance of individual nodes on the above criteria. Betweenness centrality is the fraction of all shortest paths in the network that pass through a given node and can be used to detect important anatomical or functional connections. Bridging nodes that connect disparate parts of the network often have a high betweenness centrality. BC's CV value shows a very different pattern from those of CPL and CC across levels of consciousness. Compared with the CV values of BC, the CV values of CPL and CC are significantly higher than those of EMCS at the VS/UWS level, whereas the CV of BC presents the opposite result, which is significantly lower than those of MCS and EMCS at the VS/UWS level. This suggests that The VS/UWS exhibits significantly higher variability in network integration and segregation compared to MCS and EMCS. However, the centrality of the functional network in VS/UWS remained lower. It is noteworthy that the CV values of BC are also significant in spatial properties. MCS and EMCS exhibit high node centrality variability in the centro-parietal region compared to VS/UWS. Besides, a close correlation was found between the variability of the betweenness centrality of centro-parietal electrodes and the CRS-R scores of the patients, and when the variability of CPL and CC was negatively correlated with CRS-R, only the CV of betweenness centrality was positively correlated with CRS-R. This underlines the significant role of centro-parietal regions in organizing functional networks within the conscious brain, aligning with the findings of previous network studies (24, 47).

Although the underlying mechanisms remain unclear, the variability of networks has added new and valuable characteristics in understanding the brain conditions of DOC patients. Most previous studies have concentrated on the magnitude of functional connectivity and networks in patients with DOC (12, 45). However, it is not easy to find common network properties among patients, as DOC patients have large individual differences from each other, which is impossible to balance in experiments. For example, the size and location of brain injury always change the structure of functional networks in patients after trauma. However, the variability measurement takes only the variation of the functional networks into account but disregards the network structures. Therefore, it is a more robust and repeatable feature in the brain assessment of DOC patients.

This study acknowledges certain limitations. First, we did not conduct CRS-R evaluations preceding or following each EEG recording, hindering our ability to correlate network variability with observed behaviors. Second, the sample size was still small and a healthy control was still required in the future study. Adding to this, to ensure ample time for rest, nutrition, and massage, each recording was limited to 15 min. Although we strived to regulate the various factors among patients, we have not completely eliminated natural and artificial influences. Such as, environment, lighting, temperature, meal times, family presence, nursing care, and patients' circadian rhythm (48). Thus, our study's findings warrant confirmation through multi-center cross-validation.

## Conclusion

This study analyzed the variability of neural networks on large time scales in DOC patients and revealed the relationships of network variability with patients' consciousness states. The findings revealed that VS/UWS patients had the most variable functional networks on a large time scale compared with MCS and EMCS patients. We suggest that the relative variability of large time networks may represent the underlying chaotic construction of neural circuits and variable brain conditions in VS/UWS. Overall, although further work should be conducted to examine the underlying mechanism, the findings indicate that the stability of functional networks is an important feature that should be taken seriously in assessing the brain conditions of DOC patients.

## Data availability statement

The original contributions presented in the study are included in the article/Supplementary material, further inquiries can be directed to the corresponding authors.

## Ethics statement

The studies involving humans were approved by Caregivers provided written informed consent for participation. According to the Declaration of Helsinki, this study was approved by the Ethics Committee of the Affiliated Hospital of Hangzhou Normal University [2023(E2)-HS-062]. The studies were conducted in accordance with the local legislation and institutional requirements. The participants provided their written informed consent to participate in this study.

## Author contributions

AG: Conceptualization, Formal analysis, Methodology, Resources, Software, Writing – original draft, Writing – review & editing. QW: Data curation, Investigation, Project administration, Resources, Writing – original draft, Writing – review & editing. QG: Data curation, Resources, Software, Validation, Writing – original draft, Writing – review & editing. YY: Data curation, Investigation, Resources, Validation, Writing – original draft, Writing – review & editing. XC: Formal analysis, Investigation, Methodology, Resources, Writing – original draft, Writing – review & editing. XH: Investigation, Methodology, Project administration, Resources, Writing – original draft, Writing – review & editing. YZ: Conceptualization, Formal analysis, Investigation, Methodology, Resources, Writing – original draft, Writing – review & editing.
