# Supplementary material for: Variability of large timescale functional networks in patients with disorders of consciousness
Source: Front Neurol. 2024 Feb 15;15:1283140. doi: 10.3389/fneur.2024.1283140 (PMC10905795; doi:10.3389/fneur.2024.1283140)
Supplement: Supplementary file 1 [file Data_Sheet_1.PDF]

Supplementary

In this study, the artifact removal is a crucial step. The electrooculogical activities (EOG) distribution would generate fake variance of the networks. As shown in Supplementary Figure S1, we applied a fastICA algorithm to identify the EOG artifacts as well as possible muscle activities. The artifact components were identified with information of topography, spectrum, average potential and appearance in the epochs. Generally, the blinks showed primarily frontal topography, low frequency spectrum (main power under 3 Hz) and transient activity in epochs. Eye movement shared similar temporal and spectrum characteristics with blinks, but with reverse polars of activity between bilateral frontal region. The artifact identification criteria were common across the patients. Usually 3-4 components were removed for each patient. And we also conducted a comparison between the EOG free epochs and EOG epochs after ICA cleaning. The EOG free epochs (total 20) were selected by our experienced medical doctor. The raw EOG epochs were selected with criteria that at least two distinct EOG activities in the epoch (10 s each). Then power spectrum density was estimated for each patient using the epochs (10 s) after pre-processing steps (Supplementary Figure S2).

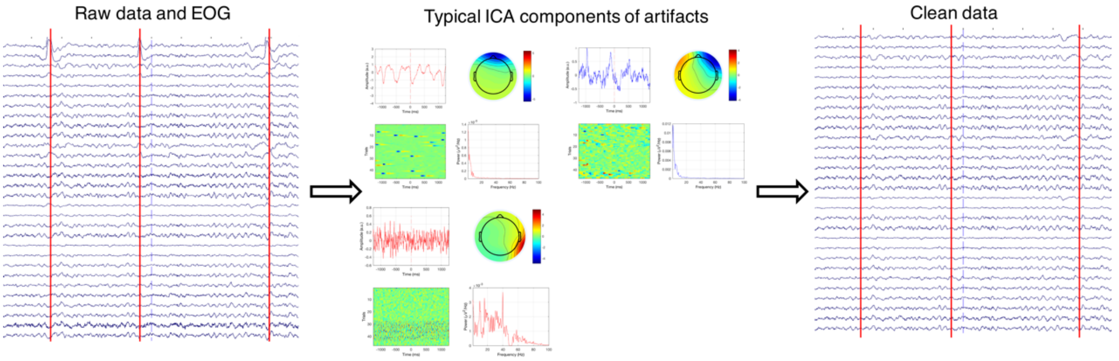

Supplementary Figure S1. Example of independent component analysis (ICA) for artifact removal. Left: an example epoch (10s) from raw EEG data of one patient with minimally conscious state. Red lines marked the electrooculogram (EOG) activities. Middle: the ICA components, which were defined as EOG and muscle activities. The artifact components were determined combining by topography, spectrum, average potential and the appearance within epochs. Right: the same epoch after ICA. Red lines show the original time points of EOG.

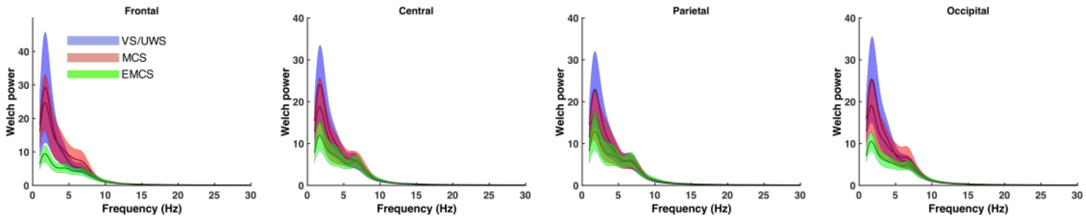

Supplementary Figure S2. Spectrum of EEG after pre-processing. The electrodes were classified into frontal, central, parietal and occipital regions according their locations.

Supplementary Table S1. Characteristics of the patients.

Number	Gender	Age	Ethology	Time since injury (months)	CRS-R score #	Diagnosis
1	Male	56	Ischemic	12	4	VS/UWS
2	Male	69	subarachnoid hemorrhage	8	5	VS/UWS
3	Male	60	subarachnoid hemorrhage	5	9	MCS
4	Male	43	traumatic brain injury	3	5	VS/UWS
5	Female	54	brainstem hemorrhage	4	18	EMCS
6	Female	62	basal ganglia hemorrhage	5	13	MCS
7	Female	48	subdural hematoma	4	14	EMCS
8	Male	44	brainstem hemorrhage	3	4	VS/UWS
9	Female	48	subarachnoid hemorrhage	5	5	VS/UWS
10	Male	40	basal ganglia hemorrhage	3	8	MCS
11	Male	56	basal ganglia hemorrhage	8	18	EMCS
12	Female	46	Ischemic	2	6	VS/UWS
13	Male	50	Ischemic	4	5	VS/UWS
14	Male	38	subarachnoid hemorrhage	3	21	EMCS
15	Female	50	subarachnoid hemorrhage	2	10	MCS
16	Male	63	brainstem hemorrhage	7	5	VS/UWS
17	Female	63	Ischemic	3	5	VS/UWS
18	Male	62	Pons hemorrhage	2	4	VS/UWS
19	Male	52	traumatic brain injury	2	9	MCS
20	Male	31	cerebral hemorrhage	3	10	MCS
21	Female	64	subarachnoid hemorrhage	3	13	MCS
22	Female	54	basal ganglia hemorrhage	2	5	VS/UWS
23	Female	49	traumatic brain injury	3	23	EMCS
24	Male	47	subdural hematoma	3	6	VS/UWS
25	Male	66	subarachnoid hemorrhage	3	6	VS/UWS
26	Male	56	subarachnoid hemorrhage	3	9	MCS
27	Male	35	brainstem hemorrhage	3	6	MCS
28	Male	65	brainstem hemorrhage	2	22	EMCS
29	Female	64	Ischemic	2	3	VS/UWS
30	Male	50	Ischemic	2	2	VS/UWS
31	Male	39	subarachnoid hemorrhage	3	10	MCS
32	Male	71	subdural hematoma	4	7	VS/UWS
33	Female	53	subdural hematoma	2	7	VS/UWS
34	Female	65	subarachnoid hemorrhage	2	21	EMCS
35	Female	41	traumatic brain injury	3	16	EMCS
36	Female	57	brainstem hemorrhage	5	12	MCS
37	Male	42	subarachnoid hemorrhage	2	6	VS/UWS

38	Male	40	traumatic brain injury	3	8	MCS
39	Male	30	traumatic brain injury	2	11	MCS
40	Male	63	subdural hematoma	2	4	VS/UWS
41	Male	79	subarachnoid hemorrhage	2	10	MCS
42	Female	53	cerebral hemorrhage	2	4	VS/UWS
43	Female	60	traumatic brain injury	2	4	VS/UWS
44	Female	80	basal ganglia hemorrhage	2	16	EMCS
45	Female	64	traumatic brain injury	2	2	VS/UWS
46	Female	63	traumatic brain injury	5	5	VS/UWS
47	Male	50	traumatic brain injury	2	15	MCS
48	Male	39	traumatic brain injury	2	15	MCS
49	Male	47	traumatic brain injury	3	23	EMCS

The CRS-R score means the best score in one week's diagnosis.
